# Open-Air "Cures" and Open-Air Work

**Published:** 1900-09-01

**Authors:** 


### OPEN-AIR "CURES" AND OPEN-AIR WORK.

Now that the public mind has become fairly broken in to the idea of the utility of open-air in the treatment of consumption, it would be well to impress upon those who are threatened with tuberculosis, as well as upon those who, by aid of sanatorium treatment, are as brands plucked from the fire, that it is as a preventive rather than as a cure that "open air" finds its greatest field of usefulness. People are so apt to regard a "treatment," or, as it is now fashionable to call it, a "cure," as a thing to be taken like a pill and be done with, and to look on all therapeutic measures as intended to restore them to their original condition and to enable them to do again the very thing that had led them into difficulties, that it is very hard to persuade them that the occurrence of their disease is not entirely due to the "infection" to which they are now taught to trace back its origin, that the fact of their being attacked by tuberculosis shows that they have a tendency that way, either from constitution or mode of life, and that the sanatorium treatment, the open air, the high feeding, and all the other things on which they are taught to pin their faith, should not be regarded as means of cure in their interpretation of the word, that is as means by aid of which they may be restored to their old work and their old surroundings, but that they ought to be regarded as means by which they may be lifted once more out of their difficulties, and be given one more chance of reforming their surroundings and leading a more healthy life. Men want to "get back to work"; that is, to get back to the mode of life which has proved disastrous to them. What to do with those who are recovering from, and with those who are threatened with, tuberculosis is a very serious problem. It is easy—at least, it is only a matter of money—to set up sanatoria for those who are ill. But these people are not ill; they are only unfitted for the work to which alone they are accustomed. They ought to be farmers, to spend their days in the open air, and to sleep in properly constructed sanitary dwellings, and that not merely for a few weeks while undergoing a "cure," but permanently, and unfortunately not much of a living is to be got out of it. It is clear, however, that if sanatoria are used merely to prop people up, and enable them to get back to the old modes of life which have produced the mischief, much of the money spent will be thrown into the gutter. The sanatorium should be the gateway opening up an open air country life, frugal perhaps, but healthy. That, however, would be a large business, and we fear unacceptable even to the recipients of the benefits it would offer.

